# Desired weight loss and its association with health, health behaviors and perceptions in an adult population with weight excess: One-year follow-up

**DOI:** 10.3389/fnut.2022.848055

**Published:** 2022-07-22

**Authors:** Cristina Bouzas, Maria del Mar Bibiloni, Silvia Garcia, David Mateos, Miguel Ángel Martínez-González, Jordi Salas-Salvadó, Dolores Corella, Albert Goday, J. Alfredo Martínez, Ángel M. Alonso-Gómez, Julia Wärnberg, Jesús Vioque, Dora Romaguera, José Lopez-Miranda, Ramon Estruch, Francisco J. Tinahones, José Lapetra, Lluís Serra-Majem, Blanca Riquelme-Gallego, Vicente Martín-Sánchez, Xavier Pintó, José J. Gaforio, Pilar Matía, Josep Vidal, Clotilde Vázquez, Lidia Daimiel, Emilio Ros, Elena Pascual-Roquet-Jalmar, Nancy Babio, Inmaculada Gonzalez-Monge, Olga Castañer, Itziar Abete, Carolina Sorto-Sánchez, Juan Carlos Benavente-Marín, Laura Torres-Collado, Marian Martin, Antonio García-Ríos, Sara Castro-Barquero, Jose C. Fernández-García, José Manuel Santos-Lozano, Cesar I. Fernandez-Lazaro, Albert Salas-Huetos, Patricia Guillem-Saiz, María Dolores Zomeño, Maria Ángeles Zulet, Amaia Goikoetxea-Bahon, Alfredo Gea, Stephanie K. Nishi, Helmut Schröder, Josep A. Tur, The PREDIMED-Plus investigators

**Affiliations:** ^1^CIBER Fisiopatología de la Obesidad y Nutrición (CIBEROBN), Instituto de Salud Carlos III (ISCIII), Madrid, Spain; ^2^Research Group on Community Nutrition & Oxidative Stress, University of Balearic Islands, Palma de Mallorca, Spain; ^3^Health Research Institute of the Balearic Islands (IdISBa), Palma de Mallorca, Spain; ^4^Department of Preventive Medicine and Public Health, Instituto de Investigación Sanitaria de Navarra (IDISNA), University of Navarra, Pamplona, Spain; ^5^Department of Nutrition, Harvard T. H. Chan School of Public Health, Boston, MA, United States; ^6^Human Nutrition Unit, Biochemistry and Biotechnology Department, Institut d'Investigació Sanitària Pere Virgili (IISPV), Hospital Universitari Sant Joan de Reus, Universitat Rovira i Virgili, Tarragona, Spain; ^7^Department of Preventive Medicine, University of Valencia, Valencia, Spain; ^8^Unit of Cardiovascular Risk and Nutrition, Institut Hospital del Mar de Investigaciones Médicas Municipal d'Investigació Mèdica (IMIM), Barcelona, Spain; ^9^Cardiometabolics Precision Nutrition Program, Instituto Madrileño de Estudios Avanzados (IMDEA) Food, Campus Excelencia Internacional (CEI UAM + CSIC), Madrid, Spain; ^10^Department of Nutrition, Food Sciences, and Physiology, Center for Nutrition Research, University of Navarra, Pamplona, Spain; ^11^Bioaraba Health Research Institute, Vitoria-Gasteiz, Spain; ^12^Osakidetza Basque Health Service, Araba University Hospital, Vitoria-Gasteiz, Spain; ^13^Department of Medicine, University of the Basque Country, Universidad del País Vasco/Euskal Herriko Unibertsitatea (UPV/EHU), Vitoria-Gasteiz, Spain; ^14^Department of Nursing, School of Health Sciences, University of Málaga-IBIMA, Málaga, Spain; ^15^Instituto de Investigación Sanitaria y Biomédica de Alicante, Instituto de Investigación Sanitaria y Biomédica de Alicante (ISABIAL-UMH), Alicante, Spain; ^16^CIBER Epidemiología y Salud Pública (CIBERESP), Instituto de Salud Carlos III (ISCIII), Madrid, Spain; ^17^Lipids and Atherosclerosis Unit, Department of Internal Medicine, Maimonides Biomedical Research Institute of Cordoba (IMIBIC), Reina Sofia University Hospital, University of Cordoba, Córdoba, Spain; ^18^Department of Internal Medicine, Institut d'Investigacions Biomédiques August Pi i Sunyer (IDIBAPS), Hospital Clinic, University of Barcelona, Barcelona, Spain; ^19^Virgen de la Victoria Hospital, Department of Endocrinology, University of Málaga, Málaga, Spain; ^20^Research Unit, Department of Family Medicine, Distrito Sanitario Atención Primaria Sevilla, Sevilla, Spain; ^21^Institute for Biomedical Research, University of Las Palmas de Gran Canaria, Las Palmas, Spain; ^22^Department of Preventive Medicine, University of Granada, Granada, Spain; ^23^CIBER Diabetes y enfermedades metabólicas (CIBERDEM), Instituto de Salud Carlos III (ISCIII), Madrid, Spain; ^24^Institute of Biomedicine (IBIOMED), University of León, Leon, Spain; ^25^Lipids and Vascular Risk Unit, Internal Medicine, Hospital Universitario de Bellvitge, Hospitalet de Llobregat, Barcelona, Spain; ^26^Department of Health Sciences, Center for Advanced Studies in Olive Grove and Olive Oils, University of Jaen, Jaen, Spain; ^27^Department of Endocrinology and Nutrition, Fundación Para la Investigación Biomedica de El Hospital Clínico San Carlos (IdISSC), Madrid, Spain; ^28^Department of Endocrinology, Institut d'Investigacions Biomèdiques August Pi Sunyer (IDIBAPS), Hospital Clinic, University of Barcelona, Barcelona, Spain; ^29^Department of Endocrinology, Fundación Jiménez-Díaz, Madrid, Spain; ^30^Nutritional Control of the Epigenome Group, Precision Nutrition and Obesity Program, IMDEA Food, Campus Excelencia Internacional (CEI UAM + CSIC), Madrid, Spain; ^31^Lipid Clinic, Department of Endocrinology and Nutrition, Institut d'Investigacions Biomèdiques August Pi Sunyer (IDIBAPS), Hospital Clínic, Barcelona, Spain; ^32^Osasunbidea, Servicio Navarro de Salud, Atención Primaria, Pamplona, Spain

**Keywords:** desired weight loss, Mediterranean diet, health perception, PREDIMED-plus trial, metabolic syndrome

## Abstract

**Background:**

Metabolic syndrome (MetS) worsens quality of life and increases mortality. Dissatisfaction with weight in patients with MetS may modify the effect of lifestyle interventions to achieve changes in health-related behaviors.

**Objective:**

To assess 1-year changes in cardiovascular risk scores, self-perceived general health and health-related behaviors according to observed changes in desired weight loss during the first year of intervention in a large cardiovascular prevention trial.

**Design:**

Prospective analysis of the PREDIMED-PLUS trial, including 5,499 adults (55–75 years old) with overweight or obesity at baseline.

**Methods:**

The desired weight loss was the difference between ideal and measured weight. Tertiles of change in desired weight loss (1 year vs. baseline) were defined by the following cut-off points: ≥0.0 kg (T1, *n* = 1,638); 0.0 to −4.0 kg (T2, n = 1,903); ≤−4.0 kg (T3, *n* = 1,958). A food frequency questionnaire assessed diet and the Minnesota-REGICOR questionnaire assessed physical activity. The Framingham equation assessed cardiovascular risks. The changes in the severity of MetS were also assessed. The Beck Depression Inventory assessed depressive symptoms and the SF-36 assessed health-related quality of life. Data were analyzed using general linear models.

**Results:**

BMI decreased at T2 and T3 (T1: 0.3, T2: −0.7, T3: −1.9). The most significant improvement in diet quality was observed at T3. Cardiovascular risk decreased at T2 and T3. Mean reductions in MetS severity score were: −0.02 at T1, −0.39 at T2 and −0.78 at T3. The perception of physical health increases in successive tertiles.

**Conclusions:**

In older adults with MetS, more ambitious desired weight loss goals were associated with improvements in diet, cardiovascular health and perceived physical health during the first year of a healthy lifestyle intervention programme. Weight dissatisfaction needs to be considered by health professionals.

**Clinical trial registration:**

http://www.isrctn.com/ISRCTN89898870, identifier 89898870.

## Introduction

Body fat excess is associated with a higher risk of several diseases (1). Prevalence of weight-related chronic non-communicable diseases is higher among subjects over 55 years of age compared to younger (2). Among non-communicable diseases, metabolic syndrome stands out, as it is a direct cause of a decrease in quality of life (3, 4) and an increase in mortality (5). Hence, obesity and metabolic syndrome should be handled to avoid such outcomes. Lifestyle factors are important aspects to consider for the management of when fat excess and metabolic syndrome should be handled. When individuals want to handle weight excess on their own, dieting and exercising are the two most widely and frequently used tools (6). Previous research has established that a Mediterranean diet (MedDiet) is a useful tool to prevent harmful cardiovascular consequences of metabolic syndrome (7). Moreover, combinations of diet and exercise are valid to ameliorate metabolic syndrome (8, 9).

However, lifestyle changes are sometimes difficult to implement, and individuals need to find their own motivation. The connection between motivators and goal pursuit was illustrated by Higgins' regulatory approach theory (10). Consequently, weight perception and weight satisfaction are more likely to trigger weight control actions than target weight status (6). A person's perception of his or her physique is referred to as body image (11). Dissatisfaction with body image can be assessed by comparing actual and declared weight (12). However, aging is associated with less concern about weight and a lower perception of weight (1, 6). If excess body fat and metabolic syndrome are ignored, health could be impaired. In addition, body image has hardly been assessed in older populations (6).

The PREvention with Mediterranean Diet (PREDIMED)-Plus trial offers a great opportunity to assess the relationship between body image dissatisfaction and health and health behaviors and health perceptions in older adults. The PREDIMED-Plus trial aims to reduce the cardiovascular consequences of metabolic syndrome through lifestyle changes (diet and exercise) (13). Although the study design ensures that all participants receive the same information and advice depending on which arm of the study they are randomized to, some participants have better adherence compared to others. In addition, body image may be an internal motivator for weight control among older adults, as it is for younger populations (6). Therefore, it is relevant to assess the role of body image in relation to the advice provided by researchers and the actual lifestyle changes observed, especially as this has been poorly evaluated in older populations (6). In this study, we will refer to body image dissatisfaction as desired weight loss (DWL) (12). Therefore, the current study aims to assess 1-year changes in cardiovascular risk scores, self-perceived general health and health-related behaviors (diet and exercise) according to observed changes in desired weight loss during the first year of intervention in a large cardiovascular prevention trial.

## Methodology

### Study design

The current study is a prospective cohort analysis of baseline and 1-year data from the PREDIMED-Plus trial. The PREDIMED-Plus trial is a randomized, multicentre and parallel-group trial that has been conducted over 6 years in 23 Spanish recruiting centers (universities, hospitals and research institutes). The aim of the trial is to evaluate the effect of combined physical activity and diet intervention on the prevention of cardiovascular disease morbidity and mortality in overweight or obese individuals. Briefly, the trial compares two interventions: an energy-reduced MedDiet with physical activity promotion and intensive behavioral support vs. usual care consisting of an unrestricted energy MedDiet (*ad libitum*) with less intensive behavioral support and no specific physical activity recommendations. The intervention in the first group focuses on healthy weight loss and weight loss maintenance, while the second group receives usual care focusing on the acquisition of healthy habits. More details on the study protocol can be found elsewhere (13) and at http://predimedplus.com/. In 2014, the trial was registered in the International Standard Randomized Controlled Trial (ISRCT; http://www.isrctn.com/ISRCTN89898870) under the number 89898870.

### Participants, recruitment, randomization and ethics

Eligible participants were community-dwelling adults. Inclusion criteria were (1) age between 55 and 75 (60–75 for women); (2) body mass index (BMI) between 27 and 40 kg/m^2^; (3) presence of at least three criteria of metabolic syndrome according to the updated harmonized definition of the International Diabetes Federation and the American Heart Association and National Heart, Lung, and Blood Institute (14). Exclusion criteria are available elsewhere (13).

Between September 2013 and October 2016, the investigators contacted a total of 9,677 individuals, of whom 6,874 were eligible to participate in the study. The included participants were randomly assigned to one of two groups in a 1:1 ratio. Randomization was stratified by center, sex and age categories. When two participants lived in the same household, they were randomized as one group. For the present work, all participants were analyzed as a cohort, and analyses were adjusted for the treatment group (see Statistical Analysis section below).

All participating institutions approved the study procedures and protocol in accordance with the ethical standards of the Declaration of Helsinki. All participants gave written informed consent.

### Body image assessment

An eating disorder questionnaire was administered at baseline and 1-year follow-up (13). The aim of the questionnaire is to detect comorbid eating disorders according to DSM-IV criteria (15). Through this questionnaire, participants were asked open-ended questions about their body perception and body image, such as ideal weight, maximum weight ever reached, current weight and height, age at which maximum weight was reached, and others not relevant for the purpose of this article. All weights were expressed in kilograms and height in meters.

Registered dietitians measured weight and height in duplicate with a calibrated high-quality electronic scale and a wall-mounted stadiometer, respectively. BMI (measured BMI) was calculated as weight in kilograms divided by the square of height in meters. Ideal BMI was calculated using the declared ideal weight and measured height. The maximum BMI was calculated as the maximum achieved weight and the measured height. Perceived BMI was calculated with declared weight and height. Perceived BMI was not classified in any way, as the purpose of the article is not the classification of perceived BMI but the accuracy of BMI. The accuracy of BMI perception was calculated by subtracting measured BMI from perceived BMI. All calculated BMIs were expressed in kg/m^2^.

At this point, a filter was applied to the dataset, excluding missing data in ideal weight at baseline or 1-year follow-up, as well as outliers in the difference between baseline and 1-year ideal BMI. Hence, the sample size was reduced to 1,375 subjects. The final sample size was of 5,499 subjects.

Body image dissatisfaction can be assessed by comparing actual weight and reported desired body weight (12). In this study, DWL was calculated at baseline and 1-year follow-up. DWL was the difference between ideal weight and measured weight, and it was expressed in kilograms. Positive values represented a desire to lose weight. Negative values represented a desire to increase weight. Tertiles were determined based on the change between 1-year follow-up and baseline. Due to the closeness of clinically relevant values (P33 = −0.5 kg; P66 = −4.4 kg), cut-off points for tertiles were rounded, so results are more easily interpreted and consistent with everyday clinical practice. Therefore, tertile 1 (T1) included all values over and including 0.0 kg (*n* = 1,638); T2 included values between 0.0 and −4.0 kg (*n* = 1,903); and T3 included values below −4.0 kg (*n* = 1,958). T1 comprised all participants who increased or maintained their DWL, while T2 and T3 comprised all the participants who decreased their DWL after 1 year to a lower or higher extent.

### Dietary assessment

Registered dietitians assessed dietary intake at baseline and 1-year follow-up with a previously validated 143-item semi-quantitative food frequency questionnaire (FFQ) (16). For each item, a regular portion size was established, and nine frequencies of consumption were available, ranging from “never or almost never” to “≥6 times/day”. Energy and nutrient intakes were obtained by multiplying the frequency by the serving size. A computer programme based on information from Spanish food composition tables was used for this purpose (17, 18). For the assessment of micronutrient intake, dietary supplements declared in the FFQ were considered.

### Determination of the dietary inflammatory index

As described by Shivappa et al., the Diet Inflammatory Index (DII) assesses the inflammatory potential of the diet (19). The DII reports the effect of 45 foods, nutrients and other bioactive compounds in the diet on six inflammatory biomarkers [four interleukins (IL-1β, IL-4, IL-6 and IL-10), C-reactive protein and tumor necrosis factor alpha]. A pro-inflammatory diet would be associated with a positive DII, while a negative DII would indicate an anti-inflammatory diet (19). The methodology for obtaining DII is described elsewhere (19, 20). Briefly, each of the 45 foods was assigned an overall inflammatory effect score. The mean standard intake of each parameter was subtracted from the individual intake of each parameter, and the result was divided by its standard deviation (SD). The centered percentile for each value was then obtained and multiplied by the overall inflammatory effect score of the dietary parameters. The sum of all dietary parameters resulted in an overall DII score.

Of the 45 DII dietary parameters, the used FFQ did not measure 15, which were not included in the DII assessment. This approach for calculating DII is available in the literature. Other authors calculated DII in this way when dietary parameters were not available (20). Therefore, the dietary parameters included were alcohol, carbohydrates, cholesterol, energy, iron, fiber, folic acid, garlic, green/black tea, magnesium, monounsaturated fatty acids, *n*−3 fatty acids, *n*−6 fatty acids, niacin, onion, protein, polyunsaturated fatty acids, riboflavin, saturated fat, Se, thiamine, total fat, trans fat, vitamin A, vitamin B12, vitamin B6, vitamin C, vitamin D, vitamin E and zinc.

### Determination of adherence to Mediterranean dietary pattern

Registered dietitians administered the 17-item MedDiet questionnaire (21) to assess adherence to MedDiet. It is a modified version of the validated questionnaire used in the PREDIMED trial (22). Each of the 17 items was related to a healthy Mediterranean dietary habit. Adherence to each dietary item was scored 1 if it was adhered to, otherwise it was scored 0. Consequently, the 17-item MedDiet questionnaire provided a score ranging from 0 to 17.

### Determination of the physical activity

Physical activity and sedentary behaviors were assessed using the validated Minnesota-REGICOR brief physical activity questionnaire (23) and the validated Spanish version of the Nurses' Health Study questionnaire (24). The metabolic equivalent of the task (MET) was calculated by multiplying the minutes spent in each activity by the intensity of the activity (light: <4.0 MET; moderate: 4–5.5 MET; vigorous: >6.0 MET) (25).

### Health variables

Socio-demographic data (primary, secondary and tertiary level of education; marital status as married, divorced/separated, widowed, single plus religious and living alone; smoking habits as current smoker, ex-smoker and never smoker) were obtained with a questionnaire administered at baseline. Medical history and current medication were also obtained. Blood pressure was measured in triplicate with a validated semi-automatic oscillometer (Omron HEM-705CP, The Netherlands) in a seated position. Fasting overnight blood samples were collected and analyzed in local laboratories using standard enzymatic methods. Fasting plasma glucose, total cholesterol, HDL-cholesterol and triglycerides were measured. Trained dietitians or nurses assessed abdominal obesity by measuring waist circumference in duplicate with an anthropometric tape, midway between the last rib and the iliac crest.

### Determination of the index to assess cardiovascular risk

Two indices were used to assess participants' cardiovascular risk: the metabolic syndrome severity score (MetSSS) and the Framingham cardiovascular risk score (Framingham Score). Both were calculated at baseline and 1 year, as previously described by Wiley et al. (26) and Peter et al. (27), respectively. The MetSSS is derived from the following parameters: waist circumference, blood triglycerides, HDL-cholesterol, blood pressure (systolic and diastolic) and glucose, whereas the Framingham score is derived from age, total cholesterol, HDL-cholesterol, blood pressure (systolic and diastolic), the presence of diabetes and smoking status.

### Determination of depressive symptoms

The validated Beck Depression Inventory II (BDI-II) was the tool used to assess the presence of depressive symptoms at both time points (28). The BDI-II is a questionnaire consisting of 21 multiple-choice questions. Each multiple-choice response receives a single score. Adding the 21 single scores gives an overall score, ranging from 0 to 63. The higher the score, the greater the severity of depressive symptoms.

### Determination of health-related quality of life

Health-related quality of life (HRQoL) was assessed using an adapted version of the SF-36 HRQoL questionnaire, validated in the Spanish population (29). It measures self-perceived HRQoL and divides perceived health into eight subscales (physical functioning, physical role, bodily pain, general health, vitality, social functioning, emotional role and mental health). In the current article, HRQoL was analyzed in two domains: physical health and mental health, which result from the grouping of the eight subscales described above (29).

### Statistical analyses

Data are shown as unadjusted mean and standard deviation (SD). Prevalence is shown as sample size and percentage. The entire sample is analyzed as a cohort. No differences were made in terms of treatment group; however, longitudinal analysis was adjusted for treatment group to avoid interactions with different treatments. Baseline analyses of descriptive characteristics were performed with one-way ANOVA (and Bonferroni *post-hoc* analysis) for continuous variables or χ^2^ for prevalence.

Changes over 1 year in body image and health (health behaviors, health perceptions and health indices) according to the tertiles described above were examined using the generalized linear model (GLM). The interaction effect was analyzed using a repeated measures ANCOVA with two factors: time (baseline vs. 1 year) as a repeated measure, groups (three tertiles) and their interactions, with sex and intervention group as covariates. The analysis of health variables was further adjusted for changes in BMI between baseline and 1 year (as a continuous variable). The reason the authors added the above adjustment for health-related variables was 2-fold (1). All variables analyzed in **Table 2** were significantly different between tertiles. (2) DWL could be affected by changes in BMI, so a reduction in weight could be a confounding factor for the analysis. The Bonferroni *post-hoc* test was performed to compare the effects of each group within and between groups. In addition, the authors assessed differences between groups at 1 year (dependent variable) after further adjustment for baseline values of the same variable (data not shown). All tests were two-tailed, and significance was set at *P* < 0.05. Analyses were performed with the statistical software package SPSS version 28.0 (SPSSS Inc., Chicago, IL, USA).

## Results

Table 1 shows the baseline socio-demographic characteristics of participants in the DWL change tertiles. Sex distribution and treatment group assignment differed between tertiles. The higher the tertile of DWL change, the higher the proportion of participants assigned to the low-calorie MedDiet group. Age, education, marital status and smoking status were similar between tertiles. Of the metabolic syndrome components, only abdominal obesity was not similarly distributed between tertiles. Baseline BMI was different between the groups, with the highest BMI found in the third tertile. The reported maximum BMI and the difference between baseline and maximum BMI were also different between tertiles. The difference between baseline and maximum BMI increased as tertiles decreased (T1: 1.9 kg/m^2^; T2: 1.6 kg/m^2^; T3: 1.4 kg/m^2^).

**Table 1:** Baseline sociodemographic and clinical characteristics according to tertiles of change of desired weight loss (DWL).

	**T1 increase in DWL or no change in DWL (*****n*** = **1,638)**	**T2 decrease in DWL up to 4.0 kg (*****n*** = **1,903)**	**T3 decrease in DWL over 4.0 kg (*****n*** = **1,958)**	***p*** **-value ‡**
	**Mean (SD)**	**Mean (SD)**	**Mean (SD)**	
Basal age (years)	65.0 (5.0)	65.1 (4.8)	64.8 (4.9)	0.228
Basal BMI (kg/m^2^)	32.5 (3.5) ^a, b^	31.8 (3.3) ^a, c^	33.2 (3.4) ^b, c^	<0.001
Maximum BMI (kg/m^2^) †	34.4 (4.1) ^a^	33.4 (3.9) ^a, c^	34.6 (4.1) ^c^	<0.001
Difference basal vs. maximum BMI (kg/m^2^) †	1.9 (2.4) ^a, b^	1.6 (2.0) ^a, c^	1.4 (2.4) ^b, c^	<0.001
Age maximum BMI (years) †	58.1 (10.4)	58.3 (10.2)	58.6 (10.2)	0.361
	*n* (%)	*n* (%)	*n* (%)	
Sex (female)	782 (47.7)	971 (51.0)	889 (45.4)	0.002
Intervention group (hypocaloric MedDiet)	535 (32.7)	885 (46.5)	1,279 (65.3)	<0.001
**Education level**				0.070
Primary	851 (52.0)	910 (47.8)	950 (48.5)	
Secondary	448 (27.4)	558 (29.3)	596 (30.4)	
Tertiary	339 (20.7)	435 (22.9)	412 (21.0)	
**Marital status**				0.457
Married	1,249 (76.5)	1,435 (75.8)	1,535 (78.5)	
Divorced/separated	128 (7.8)	155 (8.2)	137 (7.0)	
Widower	173 (10.6)	215 (11.4)	189 (9.7)	
Other (single + religious)	82 (5.0)	88 (4.6)	95 (4.9)	
Living alone ‡	202 (12.3)	263 (13.8)	224 (11.4)	0.079
**Smoking habit**				0.631
Current smoker	214 (13.1)	222 (11.7)	250 (12.8)	
Former smoker	716 (44.0)	828 (43.7)	836 (42.8)	
Never smoked	699 (42.9)	846 (44.6)	866 (44.4)	
**MetS components**				
High blood pressure	1,527 (93.2)	1,732 (91.0)	1,798 (91.8)	0.053
Hyperglycemia	1,257 (76.7)	1,433 (75.3)	1,468 (75.0)	0.436
Hypertriglyceridemia	895 (54.6)	1,092 (57.4)	1,090 (55.7)	0.248
Low HDL-cholesterol	725 (44.3)	818 (43.0)	816 (41.7)	0.295
Abdominal obesity	1,574 (96.1)	1,793 (94.2)	1,909 (97.5)	<0.001

*DWL, Desired weight loss; SD, Standard deviation; BMI, Body Mass Index; MedDiet, Mediterranean Diet; MetS, Metabolic Syndrome; HDL-cholesterol, High-density lipoprotein cholesterol. ‡ Living alone regardless of marital status. Subjects excluded from the analysis due to missing data: † T1: 9 subjects. T2: 5 subjects. T3: 4 subjects. † T1: 21 subjects. T2: 18 subjects. T3: 13 subjects. ‡ Difference in means between groups was tested by one-way ANOVA and Bonferroni's post-hoc (expressed by the letters a, b and c). Differences in prevalence across groups were examined using χ^2^*.

Changes in body image at baseline and 1 year follow-up in the DWL tertiles are available in Table 2. All parameters studied were different between tertiles at both time points analyzed and also changed differently during the period analyzed (all *p* < 0.001). In addition, all parameters were also different between tertiles at 1 year after baseline-adjusted analysis for each parameter (all *p* < 0.01). Baseline DWL was higher in tertile 3 than in the other tertiles, but at 1 year follow-up, tertile 1 had the highest DWL and tertile 3 the lowest. Thus, mean changes in DWL were −3.6 kg, 2.1 kg and 8.6 kg for tertiles 1, 2 and 3, respectively. As tertiles increased, baseline ideal BMI was lower, while BMI at 1 year was higher. Participants in tertile 1 decreased their ideal BMI at 1 year, while tertile 2 remained stable and those in tertile 3 increased their ideal BMI. Changes in measured weight were directly proportional to changes in DWL. Tertile 3 was the most successful in reducing their measured BMI after 1 year (−1.9 kg/m^2^), followed by tertile 2 (−0.7 kg/m^2^) and finally tertile 1, which had a slight increase in BMI after 1 year (0.3 kg/m^2^). Naturally, changes in weight corresponded with changes in BMI. Although perceived BMI followed the same pattern as measured BMI, the accuracy of BMI perception did not. All groups showed an accurate perception of BMI at baseline but underestimated their BMI at 1-year follow-up. Perceptual accuracy after 1 year was worse in tertile 1 than in the other tertiles.

**Table 2:** Body image (real and perceived) at baseline and 1-year follow-up according to tertiles of change of DWL.

		**T1 increase in DWL or no change (**≥**0.0 kg) (*****n*** = **1,638)**	**T2 decrease in DWL (up to 4.0 kg) (*****n*** = **1,903)**	**T3 decrease in DWL (over 4.0 kg) (*****n*** = **1,958)**	Time* **group**
		**Mean (SD)**	**Mean (SD)**	**Mean (SD)**	
DWL (kg)	Baseline	11.8 (7.5) ^b^	12.0 (6.6) ^c^	17.1 (7.8) ^b,c^	<0.001
	1 year	15.4 (8.4) ^a,b^	10.0 (6.7) ^a,c^	8.6 (6.8) ^b,c^	
	Δ	3.6 (3.8) *^d,e^	−2.1 (1.2) *^d,f^	−8.6 (4.2) *^e,f^	
Percentage DWL	Baseline	13.2 (7.2) ^a,b^	14.0 (6.4) ^a,c^	18.9 (7.1) ^b,c^	<0.001
	1 year	17.1 (7.8) ^a,b^	11.7 (6.7) ^a,c^	9.6 (7.0) ^b,c^	
	Δ	3.9 (4.1) *^d,e^	−2.3 (1.5) *^d,f^	−9.2 (4.5) *^e,f^	
Ideal BMI (kg/m^2^)	Baseline	28.1 (2.3) ^a,b^	27.2 (2.2) ^a,c^	26.8 (2.3) ^b,c^	<0.001
	1 year	27.0 (2.3) ^a,b^	27.3 (2.2) ^a,c^	28.2 (2.4) ^b,c^	
	Δ	−1.1 (1.6) *^d,e^	0.1 (1.0) *^d,f^	1.3 (1.9) *^e,f^	
Measured weight (kg)	Baseline	86.4 (13.0) ^a,b^	83.8 (12.5) ^a,c^	89.0 (13.2) ^b,c^	<0.001
	1 year	87.1 (13.5) ^a,b^	82.0 (12.8) ^a,c^	83.9 (13.0) ^b,c^	
	Δ	0.7 (3.0) *^d,e^	−1.7 (2.5) *^d,f^	−5.2 (4.3) *^e,f^	
Measured BMI (kg/m^2^)	Baseline	32.5 (3.5) ^a,b^	31.8 (3.3) ^a,c^	33.2 (3.4) ^b,c^	<0.001
	1 year	32.8 (3.7) ^a,b^	31.1 (3.5) ^a,c^	31.3 (3.6) ^b,c^	
	Δ	0.3 (1.2) *^d,e^	−0.7 (1.0) *^d,f^	−1.9 (1.6) *^e,f^	
Perceived BMI (kg/m^2^)†	Baseline	32.6 (3.6) ^a,b^	31.8 (3.4) ^a,c^	33.2 (3.5) ^b,c^	<0.001
	1 year	32.3 (3.8) ^a,b^	30.8 (3.5) ^a,c^	31.1 (3.7) ^b,c^	
	Δ	−0.3 (1.9) *^d,e^	−1.0 (1.7) *^d,f^	−2.1 (2.2) *^e,f^	
Accuracy BMI perception (kg/m^2^)†	Baseline	0.04 (1.12)	0.01 (1.16)	−0.02 (1.19)	<0.001
	1 year	−0.52 (1.40) ^a,b^	−0.34 (1.09) ^a^	−0.20 (1.30) ^b^	
	Δ	−0.56 (1.69) *^d,e^	−0.36 (1.48) *^d^	−0.18 (1.68) *^e^	

DWL, Desired weight loss; Percentage DWL, Percentage of the measured weight that DWL represents; SD, Standard deviation; Δ, Change between baseline and 1 year; BMI, Body Mass Index. † Subjects were excluded from the analysis due to missing data (T1: 94 subjects. T2: 122 subjects. T3: 153 subjects). Data analyzed by two-way repeated-measures ANCOVA were adjusted for sex and randomization. Different letters indicate statistically significant differences (p < 0.05) between groups (a, b and c), between time (^*^) and between time^*^group interaction (d, e and f) by the Bonferroni post-hoc test (p < 0.05).

Table 3 summarizes the behaviors, perceptions and health indices according to tertiles of DWL change after adjustment for confounding factors. Although some standard deviations were large, some statistically significant differences were found. Energy intake was similarly reduced after 1 year in all groups. MedDiet adherence increased in all groups after 1 year. While at baseline, tertile 3 had the lowest MedDiet adherence than tertile 1, participants in tertile 3 tended to have the most significant increases in MedDiet adherence, while those in tertile 1 tended to have the smallest increases in adherence (*p* = 0.052). Similarly, DII was slightly anti-inflammatory at baseline in tertile 1; however, after 1 year, it is pro-inflammatory for tertiles 1 and 2, and only anti-inflammatory for tertile 3. Only tertile 3 was able to change its baseline DII to a more anti-inflammatory one after 1 year. Tertiles 1 and 2 changed to a more pro-inflammatory DII, but the pro-inflammatory change was of greater magnitude for tertile 1 (*p* = 0.048). No differences were found between groups in physical activity levels or mental health (either perceived or measured); however, all groups increased their physical activity and improved their mental health over time. Cardiovascular risk improved in all groups after 1 year. While for the Framingham risk score, no differences were found between tertiles at any time point or in the time^*^group analysis; however, when cardiovascular risk was assessed using the MetSSS, some differences were found (*p* = 0.036). The highest baseline cardiovascular risk was among participants in tertile 3; however, after 1 year, tertile 3 had the lowest MetSSS. Tertile 1 changed their MetSSS minimally, while tertiles 2 and 3 decreased their MetSSS. Perceived physical health at baseline was worse in tertile 3. However, tertile 3 was the group that most improved their perception of physical health at 1 year, while tertile 1 did not change their perception over time (*p* < 0.001). Differences between groups at 1 year for the perception of physical health were also significant in the baseline adjusted analysis (data not shown).

**Table 3:** Health behaviors, perceptions and indexes according to tertiles of change of DWL.

		**T1 increase in DWL or no change (**≥**0.0 kg) (*****n*** = **1,638)**	**T2 decrease in DWL (up to 4.0 kg) (*****n*** = **1,903)**	**T3 decrease in DWL (over 4.0 kg) (*****n*** = **1,958)**	Time* **group**
		**Mean (SD)**	**Mean (SD)**	**Mean (SD)**	
Energy intake	Baseline	2,409.9 (631.6)	2,409.7 (614.1)	2,422.8 (612.3)	0.441
(Kcal/day) 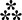	1 year	2,278.2 (484.0)	2,238.2 (489.6)	2,198.7 (477.8)	
	Δ	−131.7 (570.9)*	−171.4 (551.3)*	−224.2 (578.0)*	
17 item	Baseline	8.7 (2.6) ^b^	8.5 (2.7)	8.2 (2.7) ^b^	0.052
MedDiet Φ	1 year	11.1 (2.7)	11.7 (2.8)	12.4 (2.8)	
	Δ	2.4 (3.0)*^e^	3.2 (3.1)*	4.2 (3.3)*^e^	
Dietary	Baseline	−0.06 (2.05)	−0.01 (2.02)	0.07 (1.98)	0.048
Inflammatory	1 year	0.21 (2.01)	0.08 (2.02)	−0.25 (2.00)	
Index (DII) 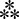	Δ	0.27 (2.08) ^e^	0.09 (2.10)	−0.32 (2.04)*^e^	
Light PA	Baseline	782.8 (947.5)	778.4 (978.2)	770.9 (923.1)	0.272
(METs) †	1 year	799.6 (923.2)	859.3 (947.2)	819.5 (939.8)	
	Δ	16.8 (1103.1)	80.9 (1127.6)*	48.6 (1082.3)	
Moderate PA	Baseline	1082.6 (1814.5)	968.7 (1431.0)	852.4 (1412.8)	0.378
(METs) †	1 year	1091.1 (1683.8)	1145.1 (1652.3)	1392.7 (1747.0)	
	Δ	8.5 (1716.0)*	176.4 (1552.4)*	540.3 (1692.3)*	
Intense PA	Baseline	804.6 (1548.4)	840.1 (1517.5)	718.2 (1309.3)	0.376
(METs) †	1 year	869.6 (1505.4)	1059.8 (1652.9)	1083.8 (1766.0)	
	Δ	65.0 (1607.4)*	219.7 (1653.4)*	365.6 (1694.1)*	
Total PA	Baseline	2670.0 (2591.0)	2587.2 (2307.7) ^c^	2341.5 (2170.5) ^c^	0.283
(METs) †	1 year	2760.3 (2379.1)	3064.1 (2457.5)	3296.1 (2588.1)	
	Δ	90.3 (2462.2)*	477.0 (2421.0)*	954.6 (2483.7)*	
Metabolic Sdr	Baseline	3.40 (1.44)	3.32 (1.41) ^c^	3.56 (1.37) ^c^	0.036
Severity Score 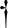	1 year	3.39 (1.45) ^a^	2.93 (1.44) ^a,c^	2.78 (1.44) ^c^	
	Δ	−0.02 (1.14)*^d,e^	−0.39 (1.18)*^d^	−0.78 (1.16)*^e^	
Framingham	Baseline	9.15 (2.98)	9.29 (2.97)	9.26 (2.94)	0.236
Risk Score 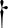	1 year	9.13 (3.02)	8.99 (3.00)	8.57 (2.96)	
	Δ	−0.03 (2.28)*	−0.30 (2.29)*	−0.69 (2.21)*	
HRQoL physical	Baseline	45.7 (8.6) ^b^	46.0 (8.6) ^c^	44.6 (9.0) ^b,c^	<0.001
dimensions 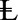	1 year	45.5 (9.1) ^a^	47.2 (8.7) ^a,c^	47.0 (8.7) ^c^	
	Δ	−0.2 (7.9) ^d,e^	1.2 (7.9)*^d,f^	2.4 (8.2)*^e,f^	
HRQoL mental	Baseline	50.9 (10.7)	51.3 (10.0)	51.8 (10.3)	0.680
dimensions 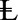	1 year	51.5 (10.2)	51.4 (9.7)	51.9 (9.7)	
	Δ	0.6 (10.3)	0.1 (10.0)	0.1 (10.4)	
BDI-II †	Baseline	8.5 (7.5)	8.2 (7.3)	8.4 (7.2)	0.864
	1 year	7.4 (7.2)	6.6 (6.6)	6.4 (6.7)	
	Δ	−1.2 (6.3)*	−1.6 (6.1)*	−2.0 (6.2)*	

*DWL, Desired weight loss; SD, Standard deviation; Δ, Change between baseline and 1 year; DII, Dietary inflammatory index; 17 item MedDiet, 17-item Mediterranean dietary questionnaire; PA, Physical activity; HRQoL, Health-related quality of life (SF-36); BDI-II, Beck Depression Inventory-II; † Measured in MET (Metabolic equivalent of task) min/week. Subjects excluded from the analysis due to missing data: 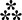 T1: 37 subjects. T2 38 subjects. T3 31 subjects. Φ T1: 5 subjects. T2 6 subjects. T3 7 subjects. † T1: 2 subjects. T2 1 subjects. T3 2 subjects. 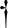 T1: 290 subjects. T2: 315 subjects. T3: 329 subjects. 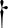 T1: 314 subjects. T2: 373 subjects. T3: 345 subjects. 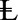 T1: 141 subjects. T2: 148 subjects. T3: 120 subjects. † T1: 17 subjects. T2: 12 subjects. T3: 9 subjects. Data analyzed by two-way repeated measures ANCOVA were adjusted for sex, randomization and change in BMI between 1-year follow-up and baseline. Different letters indicate statistically significant differences (p < 0.05) between groups (a, b and c), between time (^*^) and between time^*^group interaction (d, e and f) by the Bonferroni post-hoc test (p < 0.05)*.

## Discussion

The current study shows that in overweight older adults, high DWL has a positive impact on lifestyle, health and perceived quality of life in the first year of a healthy lifestyle promotion programme. The decrease in desired weight loss occurred simultaneously with improvements in diet, cardiovascular health and perceived physical health.

Body image can be defined as an individual's perception of his or her own body, body shape or BMI (11). It is not only limited to the perception of the body (perceptual component), but also has a cognitive component, as it includes the related feelings, and a behavioral component, in the actions taken because of the other two components (30). Body image distress may contribute not only to weight stigma but also to eating disorders (30). Body image is a multidimensional construct that is difficult to simplify (31). Several tools are available to assess body image. Dissatisfaction with body image can be assessed by comparing actual and reported weight (12). In this study, the authors used the DWL as a simplistic approach to body image perception/dissatisfaction, with the aim of using tools readily available in everyday clinical practice.

The present results are consistent with those of Jung et al. (32), who described that higher DWL was related to higher BMI in an obese adult population. DWL is a measure of dissatisfaction with body weight (12), and dissatisfaction with weight was related to increased BMI (33). Furthermore, lower weight concern is a predictor of greater long-term weight loss (34). Consequently, high-weight loss expectations at baseline have a negative effect on actual weight loss and attrition (35). Similarly, in our sample, participants who increased their DWL after 1 year showed a slight increase in their actual weight and BMI, whereas participants who decreased their DWL reduced their weight and BMI. On the other hand, previous research has described that, although changes in DWL tend to be greater among younger adults compared to older adults, older adults are more adaptive to weight gain (36). Furthermore, Grave et al. (37) described that greater DWL at the start of a weight loss programme was related to greater prior maximal weight loss. Discrepancies with baseline DWL in our sample could be explained by the fact that we used BMI instead of weight alone to assess maximum weight.

In younger adults, DWL was not related to the accuracy of BMI perception (38). However, in our sample (of older adults), those who reported an increase or no change in DWL underestimated their BMI more after 1 year. Consequently, that group gained some weight during the period studied. No differences were found in the accuracy of weight perception after 1 year between the DWL decrease groups. Accuracy in estimating body image increases after a weight loss programme (39), although there is a tendency to underestimate body weight over time (40). Unfortunately, an accurate perception of being overweight does not lead to a lower BMI (33).

A recent review on body satisfaction and depression has linked greater body dissatisfaction with an increased likelihood of depression (41). Furthermore, the detrimental effect of excess weight on mental health depends on the perception of weight (42, 43). Indeed, underestimation of excess weight was associated with lower rates of depression (44). These findings were simply supported by cross-sectional evidence. No differences were found between DWL tertiles in either depression or perceived mental health; however, depression decreased after 1 year. This could be explained by the fact that BMI perception also decreased after 1 year in all groups. Weight perception has previously been related to wellbeing (45). This could be mediating the decrease in depression questionnaire scores observed in our sample. In addition, weight stigma has been inversely related to perceived mental health (46). Greater internalization of weight stigma was related to greater DWL (32). Therefore, it is likely that weight stigma mediates the association between mental health and DWL.

Among overweight people, weight loss has been associated with improved perceptions of physical health, but not with perceptions of mental health (4). However, our analysis was adjusted for changes in BMI, so the change in perceived physical health in our case might not be mediated by a decrease in BMI. On the other hand, obesity is often perceived as a threat to health, causing diseases, such as metabolic syndrome or cardiovascular damage. The threat is strongly perceived after experiencing diseases related to excess weight (47). Therefore, BMI perception might have played a role in the perception of physical health, as changes in health perception and BMI were parallel in our sample. Consequently, weight perception and health are strong motivators for weight loss, the latter being the most important among older people (48, 49). Furthermore, a decrease in MetSSS occurred simultaneously with an increase in health perception (tertiles 2 and 3), while no change in health perception and MetSSS were also simultaneous (tertile 1). Previous research has linked improved health perception to a metabolically healthy profile in normal-weight individuals (50). Furthermore, in Mediterranean older adults, metabolic syndrome has a negative effect on the perception of physical health (3). Moreover, perceived overweight has been associated with an increased 10-year risk of cardiovascular events, even after adjustment for body composition (51). Changes in cardiovascular risk, together with a perceived threat (48, 49), had an impact on the perception of physical health.

Changes in physical health could be related to lifestyle changes, such as diet and physical activity. Physically active young Mediterranean adults have been reported to have a more accurate weight perception and to be more satisfied with their body image (52). In the current population of older Mediterranean adults, a cross-sectional study has described an inverse relationship between DWL and physical activity (53). These associations were described in other populations and a systematic review (54). In contrast, De Araújo et al. (55) described a lack of relationship between physical activity and weight satisfaction in adults. Reductions in physical activity over time were related to weight regain, in contrast, to baseline physical activity levels (56). Fortunately, in the current sample, all groups increased their physical activity over time, with tertile 3 showing the largest increase in magnitude. However, no significant differences in changes in physical activity levels were found between tertiles. Although the results are not significant, the mean values are in line with existing literature that inversely associates DWL and weight perception with physical activity.

All tertiles reduced their caloric intake after 1 year. However, although energy intake was similar between tertiles, dietary patterns and diet quality were not. An improvement in dietary intake was associated with a decrease in DWL. The evidence on satisfaction with weight and caloric intake is conflicting (53, 57), so no conclusions can be drawn. However, the desire to lose weight compared to the desire to maintain weight was related to an increased search for lower-calorie meals (58). An increase in DWL appeared to be related to a lower increase in adherence to the MedDiet. Therefore, as the current results show, MedDiet adherence was inversely related to DWL (53). In contrast, as stated in the methodology, DII is an index obtained by combining the intake of micronutrients and some foods or condiments (19). Previous literature has linked DWL to a decrease in macronutrient intake while maintaining an adequate intake of micronutrients (57). Even if micronutrient intake meets requirements, changes in DII may occur. According to our findings, weight dissatisfaction has been linked to weight management practices, such as dieting and exercise (59, 60). In the present programme, nutrition education was provided to participants, among other interventions (13). It was suggested that nutrition knowledge improves dietary intake, although it is not sufficient to achieve a healthy diet or reduce body dissatisfaction in young adults (61). Consequently, our results show that providing the same advice for all tertiles and adjusting the analysis by treatment group, some groups improve their diet more than others. According to these results, DWL may be affecting this outcome. This is relevant because following a healthy diet is related to weight loss maintenance (62).

A possible explanation is that a high DWL at baseline led to stricter adherence to the lifestyle advice provided in the present programme (59). Better adherence led to higher levels of physical activity and better diet quality. Lifestyle changes reduced the severity of the metabolic syndrome (7), which had an impact on the perception of physical health (3). Such improvements, together with the weight lost during the intervention year, could have reduced DWL after 1 year (6). Participants felt more satisfied and healthier and were therefore satisfied with a lower DWL. In other words, the DWL as a percentage of their current weight was lower after 1 year than at baseline. In addition, the ideal BMI was higher after 1 year than at baseline.

### Strengths and limitations of the study

The strength of the current research is that it includes a large sample size from a multi-center study. In addition, the longitudinal design provides stronger evidence than cross-sectional designs. The risk of reporting bias is reduced due to the standardized protocol that was followed. Second, to our knowledge, the availability of scientific evidence addressing body image or weight dissatisfaction available in older adults is limited (6). The present research contributes to increasing the body image evidence pool for overweight older adults. Finally, the cut-off points for the tertiles were rounded from the 33rd and 66th percentile, which makes the results easier to interpret and facilitates the use of the findings in clinical practice.

However, the present study has some limitations. The main limitation is that body image is a multidimensional construct that is difficult to simplify (31). Weight dissatisfaction was obtained using surrogate parameters; however, there are other methods to assess body image (63). This method was chosen to facilitate the estimation of weight dissatisfaction in clinical practice. Second, although the study had two intervention groups, for the present investigation, the study population was considered as a single cohort. To avoid confounding factors related to the intervention, all longitudinal analyses were adjusted for the intervention group. Third, the use of self-reported questionnaires has a risk of recall bias. The socioeconomic status could not be assessed because no information was collected. In addition, weight and BMI do not allow assessment of body composition, which is strongly related to health outcomes. Unfortunately, data on body composition were not available. Fourth, the participants in the present study were older than 55 years and at high-cardiovascular risk. In addition, the two interventions applied were specifically designed to prevent cardiovascular events (13). This limits the generalizability of the findings, as they could not be applied to other weight control strategies, to younger adults or normal-weight individuals. Finally, regression toward the mean might have influenced the results; however, the differences found between tertiles support the conclusions of the present study.

## Conclusion

The current study adds to the limited evidence on body image and weight dissatisfaction available in older adults with excess weight. In older adults with MetS, more ambitious desired weight loss goals were associated with improvements in diet, cardiovascular health and perceived physical health in the first year of a healthy lifestyle intervention programme. However, if such weight loss goals are unrealistic, they may be reduced over time. Improvements in lifestyle, health and, more importantly, perception of quality of life may decrease DWL among overweight or obese individuals. Health professionals should take DWL into account when implementing interventions aimed at lifestyle improvement or weight management. A simple question about what the subject's ideal weight is can add valuable information for health professionals.

## Data availability statement

The datasets generated and analyzed during the current study are not publicly available due to data regulations and for ethical reasons, considering that this information might compromise research participants' consent because our participants only gave their consent for the use of their data by the original team of investigators. However, collaboration for data analyses can be requested by sending a letter to the PREDIMED-Plus steering Committee (predimed_plus_scommittee@googlegroups.com). The request will then be passed to all the members of the PREDIMED-Plus Steering Committee for deliberation.

## Ethics statement

Research Ethics Committee from all recruitment centers approved the study protocol, according to the ethical standards of the Declaration of Helsinki. All participants provided written informed consent. All centers have the Ethics approval and consent from all the Ethic Committee. The trial was registered at the International Standard Randomized Controlled Trial (ISRCTN: http://www.isrctn.com/ISRCTN89898870) with number 89898870 and registration date of 24 July 2014, retrospectively registered. The patients/participants provided their written informed consent to participate in this study.

## Author contributions

CB and MB conducted the statistical analyses and drafted the article. CB, MB, and JT made substantial contributions to the conception and design of the work. All authors contributed substantially to the acquisition of data or analysis and interpretation of data, revised the article critically for important intellectual content, and approved the final version to be published.

## Funding

The PREDIMED-Plus trial was supported by the European Research Council (Advanced Research Grant 2013–2018, 340918) to MA and the official funding agency for biomedical research of the Spanish government, ISCIII, through the Fondo de Investigación para la Salud (FIS), which is co-funded by the European Regional Development Fund (five coordinated FIS projects led by JS-S and JVid, including the following projects: PI13/00673, PI13/00492, PI13/00272, PI13/01123, PI13/00462, PI13/00233, PI13/02184, PI13/00728, PI13/01090, PI13/01056, PI14/01722, PI14/00636, PI14/00618, PI14/00696, PI14/01206, PI14/01919, PI14/00853, PI14/01374, PI14/00972, PI14/00728, PI14/01471, PI16/00473, PI16/00662, PI16/01873, PI16/01094, PI16/00501, PI16/00533, PI16/00381, PI16/00366, PI16/01522, PI16/01120, PI17/00764, PI17/01183, PI17/00855, PI17/01347, PI17/00525, PI17/01827, PI17/00532, PI17/00215, PI17/01441, PI17/00508, PI17/01732, PI17/00926, PI19/00957, PI19/00386, PI19/00309, PI19/01032, PI19/00576, PI19/00017, PI19/01226, PI19/00781, PI19/01560, PI19/01332, PI20/01802, PI20/00138, PI20/01532, PI20/00456, PI20/00339, PI20/00557, PI20/00886, and PI20/01158), the Especial Action Project entitled: Implementación y evaluación de una intervención intensive sobre la actividad física Cohorte PREDIMED-Plus grant to JS-S, the Recercaixa Grant to JS-S (2013ACUP00194), Grants from the Consejería de Salud de la Junta de Andalucía (PI0458/2013, PS0358/2016, and PI0137/2018), a Grant from the Generalitat Valenciana (PROMETEO/2017/017), a SEMERGEN Grant, EU-COST Action CA16112, Grants (FOLIUM, PRIMUS, SYNERGIA, and LIBERI) from the Balearic Islands Health Research Institute (IDISBA), funds from the European Regional Development Fund (CIBEROBN CB06/03 and CB12/03), and from the European Commission (EAT2BENICE_H2020_SFS2016). Fundació La Marató TV3 (project ref. 201630.10). Cristina Bouzas received a Fernando Tarongí Bauzà Grant. The funding sponsors had no role in the design of the study, in the collection, analyses, or interpretation of the data; in the writing of the manuscript, and in the decision to publish the results.

## Conflict of interest

Author JS-S reports serving on the board of and receiving grant support through his institution from the International Nut and Dried Fruit Council, and Eroski Foundation. Reports serving in the Executive Committee of the Instituto Danone Spain and on the Scientific Committee of the Danone International Institute. He has received research support from the Patrimonio Comunal Olivarero, Spain and Borges S.A., Spain. Reports receiving consulting fees or travel expenses from Danone; Eroski Foundation; Instituto Danone—Spain; and Abbot Laboratories. The remaining authors declare that the research was conducted in the absence of any commercial or financial relationships that could be construed as a potential conflict of interest. The reviewer MM-A declared a shared affiliation with the author BR-G to the handling editor at the time of review.

## Publisher's note

All claims expressed in this article are solely those of the authors and do not necessarily represent those of their affiliated organizations, or those of the publisher, the editors and the reviewers. Any product that may be evaluated in this article, or claim that may be made by its manufacturer, is not guaranteed or endorsed by the publisher.

## Abbreviations

ANOVA: analysis of variance
BDI-II: Beck Depression Inventory II
BMI: body mass index
DII: Dietary inflammatory index
DWL: desired weight loss
FFQ: food frequency questionnaire
Framingham Score: Framingham Cardiovascular Risk score
GLM: General Linear Model
HRQoL: Health-related quality of life
MedDiet: Mediterranean diet
METs: Metabolic equivalent of task
MetSSS: Metabolic Syndrome Severity Score
PREDIMED: PREvención con DIeta MEDiterránea
SD: standard deviation
T: Tertile.
